# Incorporation of Antibiotics into Solid Lipid Nanoparticles: A Promising Approach to Reduce Antibiotic Resistance Emergence

**DOI:** 10.3390/nano11051251

**Published:** 2021-05-10

**Authors:** Lide Arana, Lucia Gallego, Itziar Alkorta

**Affiliations:** 1 Department of Biochemistry and Molecular Biology, Faculty of Pharmacy, University of the Basque Country (UPV/EHU), Unibertsitateko Ibilbidea, 7, 01006 Vitoria-Gasteiz, Spain; 2 Department of Immunology, Microbiology and Parasitology, Faculty of Medicine and Nursing, University of the Basque Country (UPV/EHU), Sarriena Auzoa z/g, 48940 Leioa, Bizkaia, Spain; lucia.gallego@ehu.eus; 3 Department of Biochemistry and Molecular Biology, Faculty of Science and Technology, University of the Basque Country (UPV/EHU), Sarriena Auzoa z/g, 48940 Leioa, Bizkaia, Spain; itzi.alkorta@ehu.eus

**Keywords:** antibiotics, antimicrobial resistance, multidrug-resistant bacteria, drug delivery systems, solid lipid nanoparticles

## Abstract

Antimicrobial resistance is one of the biggest threats to global health as current antibiotics are becoming useless against resistant infectious pathogens. Consequently, new antimicrobial strategies are urgently required. Drug delivery systems represent a potential solution to improve current antibiotic properties and reverse resistance mechanisms. Among different drug delivery systems, solid lipid nanoparticles represent a highly interesting option as they offer many advantages for nontoxic targeted drug delivery. Several publications have demonstrated the capacity of SLNs to significantly improve antibiotic characteristics increasing treatment efficiency. In this review article, antibiotic-loaded solid lipid nanoparticle-related works are analyzed to summarize all information associated with applying these new formulations to tackle the antibiotic resistance problem. The main antimicrobial resistance mechanisms and relevant solid lipid nanoparticle characteristics are presented to later discuss the potential of these nanoparticles to improve current antibiotic treatment characteristics and overcome antimicrobial resistance mechanisms. Moreover, solid lipid nanoparticles also offer new possibilities for other antimicrobial agents that cannot be administrated as free drugs. The advantages and disadvantages of these new formulations are also discussed in this review. Finally, given the progress of the studies carried out to date, future directions are discussed.

## 1. Introduction

The discovery and therapeutic application of antibiotics have enabled the development of modern medicine. However, the abuse and misuse of antibiotics in medicine, animal health, and agriculture over the years have led to the emergence of multidrug-resistant (MDR) bacteria and the loss of efficacy of existing antibiotics. Antibiotics are broadly employed to treat infectious diseases. However, this intensive application also has developed some associated problems. According to World Health Organization (WHO), antimicrobial resistance (AMR) is one of the biggest threats to global health, and the world urgently needs to develop new tools and strategies to minimize this problem [1,2]. It has been estimated that this situation would lead to a scenario where infections caused by MDR bacteria could cause 10 million deaths each year by 2050. Moreover, this damage would also impact the economy, leading to a catastrophic situation whereby 2030 AMR could force up to 24 million people into extreme poverty [3].

The European Centre for Disease Prevention and Control (ECDC) has published some guidance on preventing and controlling infectious diseases and developing different strategies and action plans on AMR [4]. In these action plans, the need for new antimicrobial strategies is urgently required. Nonetheless, in the last years, pharmaceutical companies have reduced the investment and efforts to discover new antimicrobial drugs due to the financial risks that developing a new drug implies [5]. This situation urges us to find new properties for existing antibiotics to alleviate the AMR problem as we search for other novel antimicrobial strategies.

In this regard, nanoscience and nanotechnology could be a realistic solution to the AMR problem, as the development and study of drug delivery systems (DDSs) have provided new possibilities to improve the effectiveness of different therapeutic drugs for other complex diseases like cancer, autoimmune diseases or pathologies in the central nervous system [6,7,8]. Among all the developed DDSs, solid lipid nanoparticles (SLNs) are of particular interest not only due to their low toxicity but also because their production at a large scale is technically and economically feasible.

Hence, this review is focused on applying antibiotic-loaded SLNs as a promising strategy to overcome or, at least, reduce AMR. First, basic knowledge about AMR mechanisms is presented. Next, the most relevant SLNs characteristics are described so that the advantages of using antibiotic-loaded SLNs against AMR mechanisms can be examined. Then, the main processes by which antibiotic-loaded SLNs can be used to reduce AMR mechanisms have been explained based on currently published data. Furthermore, combined therapy of different antibiotics or delivery of new antimicrobials is presented. Finally, future perspectives and conclusions are discussed. For developing this review, PubMed and Web of Science databases have been analyzed, searching for papers related to antibiotic resistance in bacteria and SLNs without limiting the publication data of the papers.

## 2. Antimicrobial Resistance Mechanisms

Antimicrobial resistance mechanisms are naturally occurring processes that existed much earlier than the discovery of the first antibiotic as a drug for therapeutic purposes [9]. In fact, antibiotics play different roles in bacterial communities beyond killing competing bacteria to conquer new ecological niches. These functions include signaling and communication between the different members of the bacterial community [10]. Given the importance of these functions for the survival of bacteria, it has now been demonstrated that the production of antibiotics is very ancient (from 2 Gyr to 40 Myr ago) and consequently, antibiotic–resistance mechanisms have evolved in parallel [9]. Nonetheless, the intensive use and misuse of antimicrobial agents has accelerated the emergence of more antimicrobial-resistant bacteria or even MDR bacteria.

From a genetic point of view, antibiotic–resistance mechanisms are divided into intrinsic and extrinsic mechanisms. Both provide bacteria advantageous phenotypes to survive in hostile environments [11].

Intrinsic resistance mechanisms are inherent structural or functional characteristics universally found within the genome of a bacterial species. They are independent of antibiotic selective pressure and not related to the movement of genetic material between different organisms. Some of the intrinsic antibiotic resistance mechanisms are reduced membrane permeability, overexpression of efflux pumps, expression of antibiotic modifying enzymes, modified antibiotic-targets, or biofilm-formation capacity [11].

Extrinsic resistance mechanisms consist of all acquired or developed traits that confer resistance to antibiotics. These beneficial features can be achieved by three main processes: (i) mutations that alter the DNA sequence; (ii) genetic rearrangements; and (iii) acquisition of new genetic material from other bacteria via a process known as horizontal gene transfer (HGT). Nowadays, it is well-known that a high proportion of the bacterial genome corresponds to horizontally acquired genetic material [4]. In particular, genes encoding modified antibiotic targets, antibiotic transporters, regulators that control antibiotic transporters and those encoding antibiotic-modifying enzymes can be shared between bacteria to obtain an advantageous characteristic to survive to environmental specific pressure.

Bacterial conjugation is one of the main mechanisms by which bacteria can acquire antibiotic resistance genes through HGT [12]. Although these mechanisms are naturally occurring, the abuse of antibiotics has exercised an additional and continuous pressure that has increased horizontal gene transfer among bacteria, enhancing MDR bacteria selection [13].

**Figure 1:**
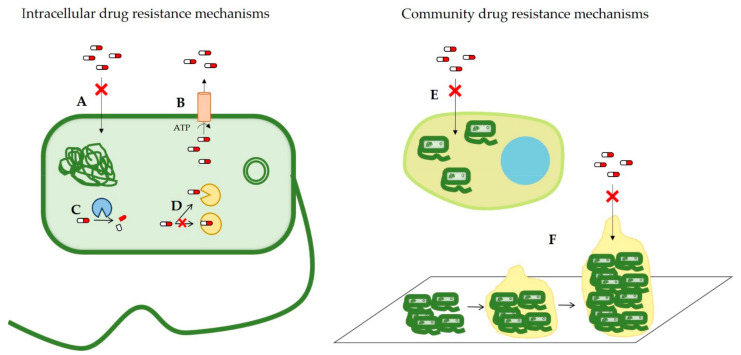
Schematic representation of the intracellular (left) or community (right) antibiotic resistance mechanisms. Intracellular antibiotic resistance mechanisms are (**A**) decreased cell wall permeability, (**B**) increased expression of efflux pumps, (**C**) expression of antimicrobial-modifying enzymes that deactivate antibiotic molecules and (**D**) modification of the antibiotic target. Community antibiotic resistance mechanisms are (**E**) intracellular infection or (**F**) biofilm-formation.

In general, the main strategies related to antimicrobial resistance mechanisms are reduction of the intracellular drug concentration thanks to a (i) decreased cell wall permeability, or an (ii) increased expression of efflux pumps; (iii) expression of antimicrobial-modifying enzymes that deactivate antibiotic molecules and (iv) modification of the antibiotic target [11]; or community-related drug resistance mechanisms, such as (v) intracellular infection [14] and (vi) biofilm-formation [15]. Antibiotic resistance mechanisms mentioned in this section are summarized in Figure 1.

### 2.1. Reduced Drug Permeability

The bacterial cell wall has a complex architecture that regulates molecular permeation to preserve cellular functions and bacterial characteristics. As a consequence, cell walls can considerably reduce the access of antibiotics to bacteria. This is especially true for anionic antibiotics, as the cell wall comprises many anionic molecules, and electrostatic repulsion impedes antibiotic penetration. Antibiotic characteristics are of particular importance when acting against Gram-positive or Gram-negative bacteria due to their different wall architecture. Gram-positive bacteria present a cytoplasmic membrane covered by a hydrophilic peptidoglycan network. Gram-negative bacteria have a more complex architecture in which the cytoplasmic membrane is surrounded by a thin peptidoglycan layer that is covered by an asymmetric outer membrane composed of phospholipids in its inner leaflet and lipopolysaccharides in the outer leaflet. The cytoplasmic membrane and the outer membrane are separated by the periplasm.

This complex structure reduces molecule trafficking dramatically. Thus, Gram-negative bacteria express some transport proteins and channels (i.e., porins) to allow the permeation of the hydrophilic molecules necessary to survive. Some antimicrobials (generally small hydrophilic ones) can penetrate bacterial cell walls taking advantage of transport proteins and porins. Consequently, bacteria have developed the capability to modify the structure or directly reduce the expression of these proteins to diminish antibiotic permeability [16]. Therefore, resistance mechanisms based on reduced permeability are especially effective in Gram-negative bacteria, decreasing dramatically therapeutic options to treat infections [16,17,18]. However, this mechanism can also be found in Gram-positive bacteria, such as vancomycin-resistant *Staphylococcus aureus* [19].

### 2.2. Overexpression of Efflux Pumps

As mentioned before, bacteria present transport proteins and some of them are efflux pumps that expel some solutes out of the cell, allowing the active regulation of their internal environment and the extrusion of toxic molecules, such as antibiotics. Efflux pumps are widespread in different organisms, and active efflux of many antibacterial agents has been well documented in many Gram-positive and Gram-negative bacteria [17]. Five different transport proteins have been identified: (i) the ATP-binding cassette (ABC) superfamily; (ii) the major facilitator superfamily (MFS); (iii) the multidrug and toxic compound extrusion (MATE) family; (iv) the small multidrug resistance (SMR) family (a subgroup of the drug/metabolite transporter superfamily); and (v) the resistance-nodulation-division (RND) superfamily. For Gram-negative bacteria, proteins belonging to the RND family are the most relevant ones, while for Gram-positive bacteria, drug efflux pumps are usually non-RND [20]. Apart from reducing the intracellular concentration of toxic molecules, efflux pumps have other important physiological functions, such as bacterial colonization, quorum-sensing control, virulence regulation, biofilm formation and other fitness responses. Therefore, regulation of efflux pump expression and activity is a complex process with several factors and mechanisms influencing such regulation. In particular, antibiotics can induce efflux pump expression, paradoxically contributing to the resistance capacity of the bacteria. Therefore, combining antibacterial drugs with efflux pump inhibitors has been proposed as an alternative strategy to overcome antibiotic resistance [20].

### 2.3. Antibiotic-Modifying Enzymes

Some bacterial strains express enzymes able to modify or destroy host tissues and deactivate antibiotics. Genes encoding these enzymes can be intrinsically part of the bacterial genome or can be acquired by HGT. Thus, bacteria expressing an antibiotic destructing enzyme acquire a weapon against the antibiotic and develop resistance. This resistance mechanism can be acquired by Gram-positive or Gram-negative bacteria [21]. One of the most known examples of antibiotic-modifying enzymes is β-lactamases [22]. These enzymes can hydrolyze and deactivate β-lactam drugs, the most widely used group of antimicrobial agents. The expression of β-lactamases is the most common resistance mechanism in Gram-negative bacteria, and the genes encoding these enzymes can be intrinsically part of the genome or acquired [23,24].

### 2.4. Modification of the Drug Target

Some antibiotics target bacterial proteins and can modulate their functions, generally forcing loss or inhibition of bacterial proteins and, therefore, bacterial functions. The interaction affinity between the antibiotic and its target defines the efficiency and the efficacy of the antimicrobial treatment. Additionally, the specificity of antibiotic–protein interaction delimits the selectivity of the antibiotic. As a consequence, an antibiotic can have an effect on a wide spectrum of bacteria or can be more restrictive to a specific strain. To overcome antibiotic effects, bacteria can produce structural changes in target proteins that modify the antibiotic–protein interaction, triggering a reduction of the efficiency and/or efficacy of the drug. One of the most studied examples of drug target modification is structural changes in ribosomal subunits that lead to a partial or complete reduction of the antibiotic binding. Ribosomes are important antibiotic targets, as their structure is different from eukaryotic ribosomes, thus, allowing selective toxicity of antibiotics against prokaryotic cells. Unhopefully, bacteria have developed many different mechanisms that modify ribosomal structures and protect themselves against antibiotic effects [25]. One of those strategies is the methylation of the ribosome in the target site so that the antibiotic cannot exert its activity, and this mechanism can be observed in both Gram-positive or Gram-negative bacteria [21].

### 2.5. Intracellular Infection

Some clinically relevant bacteria, such as *Mycobacterium tuberculosis*, *Salmonella enterica*, *Chlamydia trachomatis* and *Listeria monocytogenes*, called intracellular pathogens, can invade host cells, multiply inside them and modulate host cell biology [14]. This mechanism provides protection against antibiotics and against the host immune system. As a consequence, these pathogenic bacteria (Gram-positive or Gram-negative) can disseminate and infect different tissues in the body [14]. When bacteria invade eukaryotic cells, a new challenge is added to the previously mentioned difficulties in drug distribution, as the plasma membrane is an impermeable barrier to small and polar drugs. Therefore, intracellular infections are difficult to eradicate because bacteria are protected against antibiotic drugs, as optimal intracellular drug concentration is difficult to obtain. Thus, the ability to infect host cells is another AMR mechanism that should be addressed by developing new therapeutic strategies, such as developing DDSs for antibiotic administration [14].

### 2.6. Biofilm Formation

Biofilms are microbial communities surrounded by extracellular polymeric molecules attached to inert or living surfaces [26]. For developing a biofilm, planktonic bacteria transform into adherent bacteria due to a differential protein expression and extracellular matrix formation. Thus, bacteria present in biofilms exhibit different phenotypes compared to planktonic ones.

Biofilm-forming bacteria (both Gram-positive and Gram-negative) develop antibiotic resistance; owing to this inherent resistance, the treatment of biofilm-associated infections is considered a challenging task. Several mechanisms have been reported as responsible for the antimicrobial resistance in biofilm structures, such as poor diffusion of antibiotics through the biofilm matrix, physiological changes due to slow growth rate and starvation responses, phenotypic change of the cells forming the biofilm, quorum-sensing, overexpression of efflux pumps, and the presence of particularly resistant cells that avoid death when exposed to antimicrobials [27].

## 3. New Strategies to Overcome Antimicrobial Resistance Mechanisms

As mentioned before, the number of newly discovered antimicrobial drugs has been reduced in the last years. This situation, in combination with the increase in MDR bacteria, leads to an emergency situation where we are running out of effective antibiotics. Moreover, the appearance of resistant bacteria against these new drugs seems highly probable.

In this context, it has been suggested that developing new strategies to overcome bacterial resistance mechanisms is needed. Aiming to provide solutions to this problem, nanotechnology has expanded the therapeutic options of current antibiotic drugs through the design of DDSs, able to improve antibiotic characteristics, reduce AMR mechanisms and/or facilitate drug administration.

Therefore, a proper nanocarrier would avoid or reduce AMR mechanisms by increasing antibiotic effectiveness. Typically, DDSs can increase drug efficiency by (i) enhancing drug stability or solubility; (ii) improving release kinetics; (iii) performing a targeted drug delivery to a specific cell, tissue or molecule; (iv) allowing a higher drug penetration; or (v) obtaining better biodistribution. Moreover, it has been observed that nanocarriers can enhance drug accumulation near infections because blood vessels at infection sites are leaky [28]. Improving such relevant pharmacokinetics and pharmacodynamics factors can contribute to the reposition of antibiotics that nowadays are ineffective against resistant bacteria.

There is a wide repertoire of nanoparticulated options to treat each therapeutic problem by a tailored approach. Among the most studied organic DDSs, it is worth mentioning micelles, dendrimers, polymeric nanoparticles or micelles, polymer–drug/protein conjugates, and lipid nanoparticles. Several inorganic nanoparticles have also been employed for drug delivery applications, such as quantum dots, metallic nanoparticles or mesoporous silica nanoparticles [29].

Lipids are particularly promising components for DDSs since they are biocompatible molecules, able to self-assemble and mimic the structure and function of cellular structures [30]. Therefore, lipid-based nanoparticles are less toxic and more biocompatible than inorganic or polymeric nanoparticles [31]. Different nanoparticles can be obtained composed of biocompatible and biodegradable lipids: liposomes, nanostructured lipid carriers or solid lipid nanoparticles, for instance. Liposomes have been the most applied lipid-based drug delivery systems, and they have also been approved for clinical treatments [32]. Nonetheless, they present a low capacity to encapsulate hydrophobic molecules, low storage stability and eventual drug leakage in the media [31,32].

**Figure 2:**
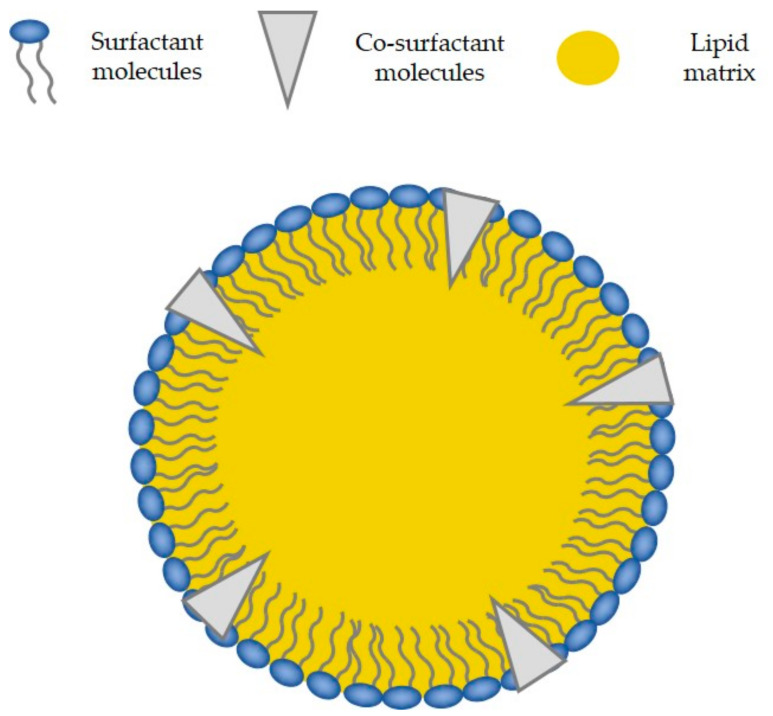
Scheme of the proposed structure of a solid lipid nanoparticle showing the solid lipid matrix, the surfactant, and the co-surfactant.

Solid lipid nanoparticles (SLNs) offer an interesting alternative to liposomes as promising robust nanocarriers for controlled drug delivery. They present a hydrophobic internal core composed of solid lipids at room or body temperature and an external stabilizing layer formed by amphiphilic surfactants and sometimes co-surfactants (Figure 2).

They present many advantages, such as low toxicity, high stability, high biocompatibility and biodegradability, and the remarkable capacity to incorporate both hydrophilic and lipophilic compounds. In addition, they enable a controlled release of the incorporated drug, and they provide chemical protection to the compound. They can be produced by simple and economical large-scale production, and it has been demonstrated that they can be effectively administrated through a wide variety of routes (oral, parenteral, rectal, nasal, ocular, etc.) [31]. The components, production methods and characterization protocols for the SLNs development are beyond the scope of this review and are already reviewed elsewhere [31,33,34,35].

Another advantage of SLNs is that their surface can be easily modified to modulate some relevant biological interactions. For instance, signaling molecules can be added to obtain targeted drug delivery, or surface characteristics can be altered by adding polymers (such as polyethylene glycol, PEG) to obtain a steal drug delivery system inside the organism [31,36]. The addition of hydrophilic molecules to the SLNs surface can also modulate the formation of the “protein corona”, i.e., the addition of plasma proteins to SLNs surface when they enter the bloodstream [37]. This protein corona formation occurs in different lipid nanoparticles (including SLNs [38]), and it has been demonstrated that it can alter nanoparticle biodistribution [37,39]. It has been demonstrated that the incorporation of PEG molecules led to a significantly reduced amount of bound proteins [40]. Thus surface modification can be a relevant advantage for systemic SLNs administration.

In summary, compared to other DDSs systems, SLNs are less toxic and more biocompatible than nanoparticles that are not composed of lipids. Moreover, they present a versatile and stable alternative to liposomes, as they present good loading capacity for both hydrophobic and hydrophilic drugs. These characteristics make SLNs suitable DDSs for antibiotic drug delivery systems, as they present good permeation through biological barriers, good cell uptake, and they can be administrated through different routes [31,32]

## 4. Relevant SLNs Characteristics for an Efficient Drug Delivery

It has been described that some SLNs characteristics influence drug stability, release kinetics, selective drug delivery or the ability of drugs to penetrate the bacteria and infected host cells, thereby increasing their efficiency. Moreover, SLN characteristics define their behavior in a biological environment. Unfortunately, it is not easy to determine the correlation between SLNs characteristics and the way they act in vivo. Thus, it is difficult to predict or extrapolate the ability of a new formulation. Therefore, a detailed characterization process is always required in each project. Nonetheless, some research has been performed to shed some light on the characteristics that SLNs need to improve antibiotic efficiency and reduce resistance mechanisms, as it is discussed in the next subsections. For SLNs formulations, the characterization of each new preparation requires at least the determination of (i) size, polydispersity and zeta-potential; (ii) solid-state and crystalline structure of the lipid core; and (iii) drug entrapment efficiency and loading capacity.

### 4.1. Size, Polydispersity and Zeta-Potential

Although different physicochemical characteristics can be evaluated, size, polydispersity and surface charge are the most relevant characteristics as they can define cell uptake, biodistribution or toxicity in vivo [32].

Nanoparticle size generally states for the mean diameter of a nanoparticle population. As it is usually determined by dynamic light scattering, nanoparticle size refers to the mean hydrodynamic diameter of the nanoparticle population. Nonetheless, these values should be confirmed by other techniques, such as electron microscopy or atomic force microscopy.

Polydispersity or polydispersity index (pdi) is a parameter that defines the size range of a nanoparticle suspension; therefore, it defines the uniformity of the size distribution of a nanoparticle population. The theoretical range of the index is 0–1, and bigger nanoparticle size-uniformity correlates to smaller polydispersity index values. To develop predictable, safe and stable DDSs, it is important to obtain homogeneous nanoparticle suspensions. Thus, small pdi values are desirable. For SLNs suspensions, pdi values of 0.3 or below are considered acceptable, indicating a homogeneous population [34,41].

Zeta-potential reflects the potential difference between the electrophoretically mobile particles and the layer of dispersant around them. It is mainly used to describe nanoparticle stability, as higher zeta-potential values refer to bigger electrostatic repulsion, and this is supposed to avoid particle aggregation and stabilize the nanoparticle suspension [42]. Thus, nanoparticles with a zeta-potential >±30 mV are considered highly stable. Nonetheless, as other interactions apart from electrostatic forces can influence particle stability (i.e., van der Waals attractive forces), it is possible to obtain stable particles with low zeta-potential values and vice versa. Zeta-potential may also be applied to assess the surface charge of nanoparticles, provided certain conditions are respected in the measuring protocol [34,42].

SLNs have been described as nanoparticles with a mean diameter range of 50–1000 nm. Different size ranges (from 85 nm to 615 nm) have been obtained with different SLNs formulations that significantly enhance the antibiotic activity of different drugs in planktonic or biofilm-forming bacteria (Table 1 and Table 2). Nonetheless, depending on the purpose of the designed SLNs, a more specific size range may be required. For instance, in systemic administration, it seems that 50–200 nm-sized nanoparticles are optimal to obtain a long circulatory condition, but this range can vary depending on nanoparticle composition and shape [41,43,44]. Moreover, SLNs larger than 500 nm present reduced access to de lymphatic system [45] and nanoparticle size also seems relevant to treat central nervous system infections, as only SLNs smaller than 200 nm seem to efficiently overpass the blood–brain barrier [41,44]. Additionally, to treat intracellular infections, it must be taken in mind that nanoparticle size can influence nanoparticle uptake mechanism by infected eukaryotic cells, and the internalization pathway can be relevant for the improvement of drug efficiency [46,47].

The nature of the cell wall can determine the interactions between SLNs and bacteria. Therefore, each SLNs composition can interact by a different pattern with different bacterial strains. In this regard, it is important to take in mind that highly efficient SLNs must interact with the cell wall to deliver the cargo into the cell. As both Gram-negative and Gram-positive bacteria are negatively charged, it has been suggested that efficient DDSs should have a positive charge to facilitate electrostatic interactions [48,49,50]. Pignatello and coworkers have reported that positive charged SLNs allowed a better interaction with the bacteria surface and an enhanced penetration through their cell wall, as the growth inhibitory activity tended to enlarge with the increasing SLNs positive charge. Among all the bacterial strains they tested, this feature was more evident against Gram-negative bacteria [49]. In a recent paper, González-Paredes and coworkers analyzed the interactions between different SLNs suspensions and bacterial walls using confocal microscopy and flow cytometry. They observed that cationic SLNs, but not anionic SLNs, displayed rapid and extensive interaction with bacterial cells, independent of their lipid core composition [50]. Interestingly, they also observed that the lipid core seemed to have an important role in determining the intracellular delivery of their cargo (an oligonucleotide), obtaining the most efficient intracellular delivery with tripalmitin-based lipid core. Nevertheless, other researchers demonstrated that the incorporation of antibiotics into anionic SLNs also improved drug efficiency [51,52,53].

Fazly Bazzaz and coworkers prepared and characterized different anionic SLNs compositions carrying *Eugenia caryophyllata* essential oil, a well-known antibacterial and antifungal substance. They observed that incorporation of the essential oil in SLNs effectively reduced the concentration required for growth inhibition and killing of microorganisms [54]. Interestingly, the formulations were less effective against Gram-positive bacteria than against Gram-negative bacteria and fungi. These differences highlight the relevance of different SLNs parameters (size, composition, morphology) on nanoparticle-microbe interactions [18].

Additionally, when the final goal is to treat an intracellular infection, the eukaryotic cell uptake of SLNs plays a key role in obtaining high intracellular drug concentrations and ensure treatment efficiency. It has been described that negatively charged SLNs are taken more efficiently by macrophages [55], are less toxic and present better antibiotic effects. Other interesting published results highlighted that SLNs size could affect more deeply than a charge to the cell uptake of the encapsulated drug in macrophages. In a noteworthy work, Xie and coworkers obtained enrofloxacin-loaded SLNs and analyzed cell uptake of the drug in a macrophage cell line (RAW 264.7). First, they analyzed SLNs suspensions with different sizes (i.e., 150–605 nm diameter), maintaining negative zeta-potentials from −3.1 to −24.9 mV. All SLNs suspensions improved intracellular drug concentration comparing to free drug incorporation, but they also observed that the intracellular concentration of the antibiotic was 1.147 μg/mg of protein for the biggest size SLNs suspension (605 nm) and 0.336 μg/mg of protein for the smallest one (150 nm). Next, they also prepared SLNs suspensions with different zeta-potentials adding different percentages of dimethyldioctadecyl ammonium chloride. They observed that the net charge and not its positivity or negativity influenced drug uptake efficiency. Finally, comparing SLNs suspensions with different characteristics, they concluded that particle size played a more important role than zeta potential for the intracellular delivery of the drug in macrophages [56].

Therefore, in view of these results, it is relevant to holistically consider the final purpose of tailored antibiotic-loaded SLNs, not only from the point of view of the infectious agent but also considering the location of the infection and the treatment attending to the condition of the patient.

### 4.2. Solid State and Crystalline Structure of the Lipid Core

As mentioned before, SLNs consist of a lipid core that presents a solid state at room or body temperature. This characteristic is relevant to achieve a controlled drug release, as the mobility of the drug in a solid lipid matrix is substantially lower than in oil [33,35]. More importantly, the quality of an SLN formulation depends on the crystal structure of the lipid matrix, as the solid state of the lipids influences particle stability and controlled drug release. For a DDS, stability and controlled drug release are central characteristics, as the main function of a nanocarrier is to improve drug stability and release kinetics.

The crystal structure of an SLNs formulation cannot be presumed because it depends on multiple factors, such as composition or production method, among others. In fact, supercooled melts (i.e., lipid structures that do not crystallize although being below their melting point) can appear, meaning that crystallization does not take place during the production process. Eventually, supercooled melts tend to crystallize in an uncontrolled manner leading to large crystal formations and particle aggregation [31]. Therefore, the solid state of the lipid matrix must be carefully determined, usually by infrared spectroscopy or differential scanning calorimetry.

In addition, depending on SLNs formulation during the SLNs formation process, lipids can form different crystal species with different stabilities. Therefore, after a period (usually during SLNs storage time), recrystallization processes can occur, leading to more stable structures [31,33]. Those possible polymorphic transitions are one of the main drawbacks of SLNs formulations, as, during the recrystallization process, physicochemical characteristics of SLNs formulations can change, and expulsion of drug molecules can occur. Consequently, apart from determining the state of the lipid matrix, SLNs stability studies must be performed to discard changes in SLNs characteristics during the storage, thus, ensuring pharmaceutical standards.

**Figure 3:**
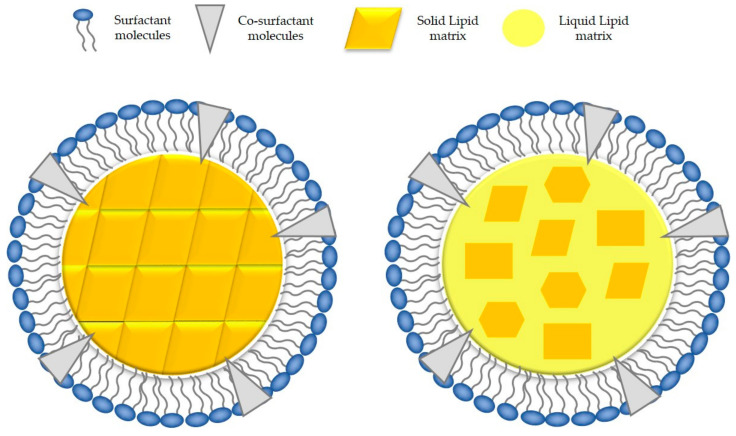
Schematic representation of lipid core structure of SLNs (**left**) and NLCs (**right**). Geometrical forms in the NLCs scheme represent different solid lipid crystals.

Given the potential complications related to the lipid crystallization process, another approach was proposed by different research groups. Liquid lipids were added to the formulations, partially replacing the solid lipid component, and nanostructured lipid carriers (NLCs) were obtained (Figure 3).

These DDSs are considered as “second-generation” SLNs (as they were presented few years after SLNs) because they do not present recrystallization processes. Thus, they avoid one of the main SLNs drawbacks [31,34,57].

### 4.3. Entrapment Efficiency and Loading Capacity of the Drug

Entrapment efficiency (EE) is defined as the percentage of drug that has been incorporated into the nanoparticle suspension compared to the total drug content in the initial formulation. Loading capacity (LC) is related to the amount of total entrapped drug divided by the total nanoparticle weight. This magnitude indicates the amount of drug delivered per amount of nanocarrier.

Both characteristics depend on the solubility of the drug in the lipid matrix, as good compatibility between the drug chemical structure and the lipid matrix ensures a good partition of the drug between the lipid and the surrounding aqueous solution. Therefore, the type and concentrations of lipids and surfactants are the most important factors that can influence drug incorporation [58].

In most of the published works, the encapsulation efficiency of different SLNs formulations is above 70% [34]. Nonetheless, it has been published that SLNs present a low loading capacity comparing to other DDSs and that this drawback could also be improved by adding liquid lipids to the lipid matrix. Thus, according to the literature, apart from presenting better storage stability, NLCs presents better drug loading capacity [31,34,57]. However, it is worth mentioning that despite being presented as a second-generation SLNs only seven years after the first publication relative to SLNs, the SLNs cover approximately 70% of the investigations carried out with lipid nanoparticles until December 2018 [34]. Thus, NLCs still needs scientific validation, and SLNs continue to be promising DDSs and remain the first choice in developing drug delivery projects. In any case, whether with the most studied SLNss at present or in the future with the NLCs, the most important aspect is to have a thorough knowledge of the potential drawbacks and limitations of these DDSs to compensate them by enhancing their advantages and adapting each DDSs to the needs of each particular project.

## 5. Solid Lipid Nanoparticle to Improve Drug Delivery

It has been broadly demonstrated that a proper DDS improves drug delivery, enhancing treatment efficiency [6,7,8]. For antimicrobial drugs, several formulations have been developed and tested to improve the antimicrobial drug effect and help to revert drug resistance processes. As mentioned before, SLNs can improve drug stability, solubility or release kinetics, but, more interestingly, to reduce AMR, they improve drug permeability and selective delivery of incorporated antibiotics.

### 5.1. Improved Permeation and Bioavailability

Some formulations can enhance antibiotic efficiency by improving bioavailability, thus, allowing oral [59,60,61,62,63,64,65,66], intramuscular [67], pulmonary [68,69], skin [70,71,72] or subcutaneous [73,74] drug administration. Other groups have demonstrated that SLNs can increase antibiotic activity by facilitating drug permeation through different barriers in the eye [75,76,77,78,79,80], through the mucosal barrier [81,82], or even through the blood–brain barrier [83].

### 5.2. Improved Selectivity

A relevant strategy to improve efficiency and reduce antibiotic resistance when administering antibiotics into an organism is developing selective drug delivery systems that target bacteria. A site-directed release of antibiotics enhances drug concentration in the infection site and reduces unspecific side effects (i.e., possible systemic toxicity), which also enables applying higher drug concentrations and reduces bacterial resistance. In this regard, some interesting works demonstrated that drug targeting by modulation of SLNs surface with selective molecules, such as antibodies, also increased loaded drug efficiency or reduced cytotoxic side effects of biocides that do not develop resistance [84]. In this context, incorporating cytotoxic biocides into SLNs can reduce cytotoxicity in eukaryotic cells while improving antimicrobial properties [85]. For instance, it has been demonstrated that incorporation of tilmicosin in castor oil-SLNs can reduce drug side effects, such as cardiotoxicity [86].

Another described strategy to obtain selective drug delivery is to develop pH-sensitive SLNs, which can tune their release kinetics depending on the pH of the environment. The aim of these formulations is to retain antibiotic drugs into the SLNs until the nanocarrier reaches the infection site where the pH is lower and, thus, the antibiotic is efficiently released in the target tissue [87,88].

## 6. Solid Lipid Nanoparticles Can Reduce Antibiotic Resistance Mechanisms

**Figure 4:**
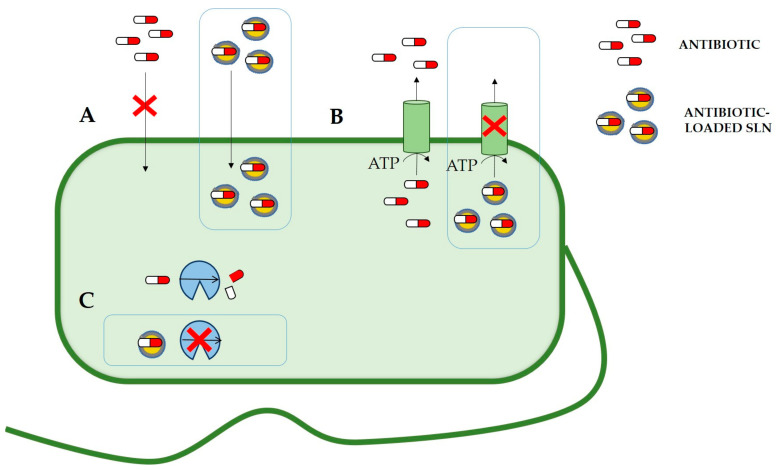
Schematic representation of the main antibiotic resistance mechanisms and the effect of antibiotic encapsulation into SLNs to avoid/reduce AMR mechanisms. Main antibiotic resistance mechanisms are (**A**) decreased cell wall permeability, (**B**) overexpression of efflux pumps and (**C**) antibiotic-modifying enzymes. The boxes depict ways to circumvent resistance mechanisms by incorporating antibiotics in SLNs. SLNs can (**A**) improve drug permeability, (**B**) reduce the activity of efflux pumps or (**C**) protect antibiotics from drug modifying enzymes.

**Figure 5:**
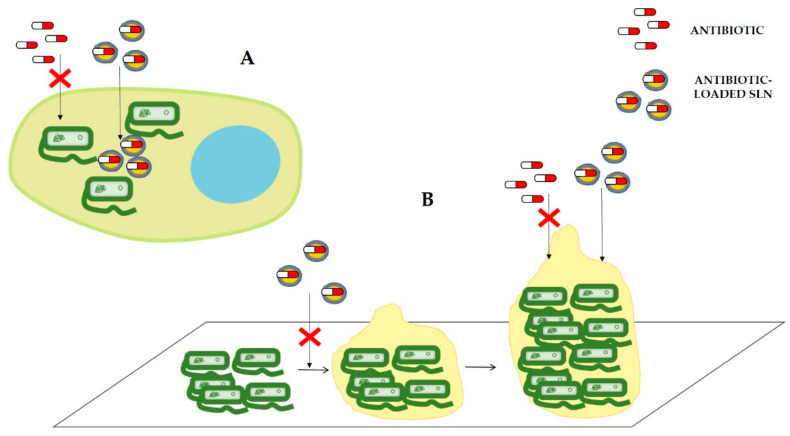
Schematic representation of community antibiotic resistance strategies and the effect of antibiotic incorporation into SLNs to avoid them. (**A**) Intracellular infection and (**B**) biofilm formation. In both cases, how can antibiotic-loaded SLNs overcome these strategies is represented. SLNs can improve eukaryotic cell uptake to treat intracellular infections. SLNs can reduce biofilm formation or viability of biofilm-forming bacteria.

As is going to be discussed in this section, several works have been published demonstrating the potential of SLNs to reduce AMR mechanisms and, thus, the multiple advantages of applying this type of DDSs to obtain an optimized formulation for in-use antibiotics. As mentioned in the previous section, the new formulations can increase the efficiency of current antimicrobial treatments by improving drug permeation, not only through different barriers in the organism but also through the bacterial cell wall. Moreover, it has been demonstrated that SLNs can (i) reduce efflux pump-mediated drug expulsion, (ii) avoid the effect of antibiotic-modifying enzymes (Figure 4), (iii) improve cell uptake to treat intracellular infections or (iv) reduce biofilm formation or viability of biofilm-forming bacteria (Figure 5).

### 6.1. Drug Efflux Pumps

One of the main strategies of antibiotic resistance is to reduce intracellular drug concentration (Figure 4). In this regard, some cells overexpress drug efflux pumps encoding genes to increase drug expulsion capacity, helping bacteria to develop resistance. According to the literature, different nanoparticle formulations can inhibit the transporter activity effectively [89].

Although many works demonstrate that SLNs enhance intracellular drug concentration [50,90], to our knowledge, only two works, in yeasts [91] and mycobacteria [92], have related the ability of SLNs to provide an effective treatment to the reduction of the drug efflux activity. Nevertheless, these authors describe the increased intracellular concentration of the antibiotic and its antimicrobial effect. Unfortunately, they have not been able to determine whether this effect is due to reduced pump activity or other reasons. This lack of mechanistic information hinders the potential optimization of DDSs. Therefore, more mechanistic information about how SLNs escape drug-efflux pumps are needed to find the best characteristics by which SLNs would increase drug effectiveness.

### 6.2. Enzymatic Degradation

As mentioned in Section 2.4, bacteria can express antibiotic modifying enzymes to inactivate antibiotics and acquire resistance. Nanocarriers can reduce this inactivation by protecting antibiotics from enzymatic activity, thus, avoiding this resistance mechanism (Figure 4) [28].

Although several publications demonstrate the capability of different organic nanoparticles to protect antibiotics from bacterial enzyme degradation (e.g., polymeric nanoparticles [93], liposomes [94] and micelle–lipid nanocapsules [93,94,95]), there is no publication, at least in our knowledge, related to the protection against enzymatic activity presented by SLNs.

Nonetheless, some works demonstrate the ability of SLNs to protect incorporated peptides from hydrolytic enzyme activities [96], but data supporting the opposite idea can also be found in the literature [97]. Therefore, the potential of SLNs to protect drugs from enzymatic degradation should be further analyzed, and more experimental results are required.

### 6.3. Infections by Intracellular Pathogens

As previously described, infections caused by intracellular pathogens are particularly difficult to treat. In this regard, SLNs are capable of interacting with lipid membranes, delivering incorporated drugs to eukaryotic cells by active or passive cell uptake mechanisms [98]. Therefore, loading antibiotics into SLNs can increase the permeation of the drug in the eukaryotic host cell, increasing antibiotic drug concentration and improving antibiotic efficiency (Figure 5).

Different works demonstrating the ability of antibiotic-loaded SLNs to increase bacteria death inside macrophages have been published [55,56,99,100]. Hosseini and coworkers obtained doxycycline-encapsulated SLNs composed of palm oil, lecithin and Tween-80 to improve drug efficiency against *Brucella melitensis*, an infection that to date has no efficient treatment because the survival of the bacteria inside the macrophages makes them safe from the immune system and disrupts drug delivery mechanism. They observed that doxycycline-loaded SLNs were significantly more effective in reducing the number of bacteria inside macrophages than free doxycycline [99]. Even more, this formulation also improved the antibacterial capacity of doxycycline to treat chronic brucellosis and preventing its relapse in vivo [101].

The ability of SLNs to interact with eukaryotic cells has a direct impact on the capability of SLNs for intracellular delivery of antibiotics. In a previously mentioned work, Xie and coworkers developed docosanoic acid-based SLNs with different properties loaded with enrofloxacin and analyzed their cellular uptake, intracellular elimination of the drug and antibacterial activity against intracellular *Salmonella* CVCC541 in RAW 264.7 macrophages. They observed that all SLNs formulations enhanced the cellular uptake of the drug and that this enhancement was significantly influenced by the size rather than by the charge (cationic or anionic). Besides, after removing the extracellular drug, eliminating SLNs-encapsulated enrofloxacin from the cells was significantly slower compared to free enrofloxacin, prolonging optimal intracellular drug concentration. More importantly, the inhibition effect against intracellular *Salmonella* CVCC541 of enrofloxacin-loaded SLNs was stronger than a free drug after all the incubation periods [56]. In a latter work, they analyzed the effect of using different saturated fatty acids as lipid matrix, developing SLNs composed of docosanoic acid (C22), octadecanoic acid (C18), hexadecanoic acid (C16), and tetradecanoic acid (C14). They observed that entrapment efficiency, loading and particle size, increased with increasing the fatty acid chain length, obtaining the best results with docosanoic acid. Specifically, all formulations presented similar cytotoxicity, but docosanoic acid-composed SLNs presented the best cellular uptake of enrofloxacin, longer intracellular retention of the drug and stronger antimicrobial efficacy against intracellular *Salmonella* CVCC541. More interestingly, they observed that fatty acid chain length influences the intracellular distribution of the drug. SLNs with longer fatty acids tend to accumulate in the perinucleus of RAW 264.7 cells, while shorter ones are mostly absorbed in the cell membrane. The authors associate these results to the higher lipophilicity of long-chain fatty acids, more than to the differences in the SLNs sizes, indicating that lipid matrix lipophilicity could be another relevant factor to take in mind to design SLNs for intracellular infections [90].

An interesting strategy to improve intracellular drug concentration is the modification of the SLNs surface to stimulate active cell uptake. In recent years, mannose has attracted some attention to treat intracellular lung infections because alveolar macrophages overexpress mannose receptors to recognize mannan-coated cell walls in organisms, such as *Mycobacterium tuberculosis*, *Streptococcus pneumonia*, *Yersinia pestis*, *Candida albicans*, *Pneumocystis carinii*, *Cryptococcus neoformans*, and *Leishmania* [102]. For this reason, mannose-receptors are interesting targets to stimulate cell uptake of antibiotic-loaded SLNs in alveolar macrophages to increase antibiotic concentration inside cells. Different groups have developed SLNs formulations modifying nanoparticle surfaces with mannose to take advantage of this mechanism [55,100]. In recent work, Ma and coworkers modified SLNs surface with mannose to activate macrophage uptake by mannose receptors, and this specific endocytic pathway improved nanoparticle cell uptake. Additionally, to improve drug retention and efficiency, the antibiotic release was pH-sensitive, obtaining a targeted drug release inside the macrophage endosomes. Combining both strategies exhibited a noteworthy increase in the intracellular antibiotic efficiency in the in vitro latent tuberculosis infection model and excellent antibiotic efficacy in the in vivo antibiotic tests compared to the free drug solution [100]. SLNs surface modification with mannose residues also has demonstrated the potential to promote selective uptake by lung tissues, obtaining a site-specific delivery and reducing side-effects [103].

### 6.4. Biofilm Formation and Quorum Sensing

Biofilms are structured communities of bacterial cells enclosed in a self-produced polymeric matrix attached to inert or living surfaces [26]. It has been demonstrated that biofilm formation presents a physical and biological barrier that reduces drug penetration and triggers drug resistance. The treatment of biofilm-associated infections is considered a challenging task, owing to their inherent resistance to (i) antimicrobial agents and (ii) the host immune system [104]. The use of nanoparticles could overcome some biofilm-related drug resistance mechanisms mentioned in Section 2.6, such as decreased drug uptake, increased efflux of the drug or reduced biofilm permeation (Figure 5).

Several works have confirmed the improvement of drug efficiency loading antibiotics into SLNs to treat infection caused by biofilm-forming bacteria [105,106,107,108]. Fazly Bazzaz and coworkers demonstrated that cationic SLNs formulations were more effective in biomass reduction of *Staphylococcus epidermidis* biofilms than the free drug form [106]. However, from their results, it can be deduced that empty SLNs could also reduce biofilm mass. Nonetheless, they demonstrated that empty SLNs showed no remarkable reduction of biofilm-embedded bacterial viability, while rifampin-SLNs significantly reduced biofilm-forming bacterial viability, improving free drug efficiency [106]. In addition, encapsulation of clarithromycin in SLNs has demonstrated important improvements in drug characteristics. Clarithromycin-containing SLNs present enhanced in vitro antibacterial activity in *S. aureus*, higher potential in biofilm eradication than free drugs, and an almost 5-fold improvement in relative oral bioavailability in rats [107].

Interestingly, it has been demonstrated that although some SLNs formulations do not improve drug efficacy against planktonic bacteria, they can be very useful to increase the antibacterial activity of the same drug in the same biofilm-forming bacterial strain [109,110]. This is another example of how difficult the extrapolation of an in vitro characteristic to its in vivo effect can be.

Bacteria use a cell-to-cell communication system known as “quorum sensing” (QS) to coordinate group behavior, such as the production of virulence factors or biofilm forming in response to adverse environmental conditions [111]. In this context, eradication of this communication can be very helpful to reduce virulence factors. In an innovative approach, Nafee and coworkers incorporated a QS inhibitor that inhibits pyocyanin formation because pyocyanin concentration has been related to the virulence level of *Pseudomonas aeruginosa* infection in cystic fibrosis. They incorporated the inhibitor into different SLNs formulations (optimized to penetrate the mucus barrier of the lung), and after characterizing them, they notably observed that inhibitor-loaded SLNs presented an improved anti-virulence activity compared to the free compound. Surprisingly, they also observed that empty SLNs presented anti-virulence activity without affecting bacterial viability, and they discovered that this activity was related to the emulsifiers they had applied (Tween-80 and Poloxamer 407) [112]. This fact is concordant with the results obtained by Fazly Bazzaz and coworkers, where they observed that empty SLNs composed of Poloxamer 188 and Tween-80 presented the capability to reduce biofilm mass [106]. Although this effect was no further analyzed by the authors, as they applied the same surfactants and observed similar anti-biofilm activity, it can be thought that surfactants may have a bioactive function in biofilm formation also in this biofilm system. These findings highlight the relevance of SLNs components and that the pharmacological activity of excipients should not be underestimated.

The main characteristics of different SLNs formulations developed to reduce antibiotic resistance mechanisms are summarized in Table 1.

## 7. Nanoparticles for Drug Combination Strategy

In complex infections, such as in multi and pan-resistant bacterial infections, combining different drugs is the only alternative that can produce any promising result. Combining more than one antibiotic can lead to an additive or synergistic effect that reduces the capacity of bacteria to endure drug activity. Nonetheless, this strategy has some drawbacks and difficulties. Each drug usually presents different pharmacodynamic and pharmacokinetic properties, and sometimes it is difficult to obtain the same spatial-temporal biodistribution for both drugs [113].

To achieve this, nanoparticles are very useful for combined therapy because incorporating different drugs in the same nanoparticle ensures the presence of both molecules simultaneously in the same place. Different works have been published demonstrating the ability of SLNs to perform an efficient drug combination [114,115,116,117]. In this context, a combination of two different antibiotics can increase the success of treatment. For instance, combining tilmicosin and florfenicol in anionic SLNs enhanced their therapeutic efficacy in different in vitro studies with bacterial strains and this improvement was also observed in in vivo experiments done in rats [118] and pigs infection models [2].

Antibiotics can also be combined with molecules of different nature. For example, clotrimazole and alpha-lipoic acid-loaded cationic SLNs have been obtained to efficiently treat topical infections of *C. albicans* [119]. Ampicillin has also been combined with curcumin, a phytochemical derived from the rhizome of *Curcuma longa* that has antibacterial and antibiofilm activity. Curcumin was found to restore bacterial susceptibility to antibiotics by inhibiting its biofilm growth mode and rendering it sensitive to antibiotics in vitro [120]. Combining ampicillin and curcumin in SLNs increased antibiotic effect in different bacterial strains (i.e., *Bacillus subtilis* and *Corynebacterium diphtheriae*) and in some other cases (i.e., *B. subtilis* and methicillin-resistant *S. aureus*), antibiotic resistance was broken [121]. Rodenak-Kladniew and coworkers demonstrated that hybrid formulations mixing lipids and chitosan (as cationic biopolymer) to obtain ofloxacin and eugenol co-loaded SLNs could improve ofloxacin efficiency as well as sustained and localized drug release, both in vitro and in vivo [122]. Antibiotics can also be combined with bioactive lipids against biofilm formation [123], with antibacterial peptides obtaining synergistic or additive effects [124], loaded into SLNs with silver complexed lipids to obtain an enhanced effect [125], or combined with lipids conjugated to selenium [126]. Antibiotics can also be combined with other molecules presenting interesting biological activities, i.e., wound healing factors [127].

Enzymes can also be used for drug combination strategies. For instance, DNase can be applied to reduce biofilm formation and increase the antimicrobial activity of the formulation [128,129]. For example, anionic lipid nanoparticles combining DNase and levofloxacin showed strong antibacterial activity against Gram-positive (*S. aureus*) and Gram-negative (*P. aeruginosa*) bacteria [128].

The main characteristics of applied SLNs formulations for combined therapy are summarized in Table 2.

## 8. Solid Lipid Nanoparticles for the Delivery of New Antibiotic Agents

In general, nanoparticles are useful tools to improve drug characteristics and enhance the efficiency of well-known antibiotics. Nonetheless, nanoparticle potential goes beyond the mentioned possibilities as they can be applied to deliver molecules that present interesting and promising biological effects but do not exhibit good characteristics of those attributed to pharmaceuticals. For instance, they do not obey Lipinski’s rules and are still under development. Here we describe the incorporation in SLNs of oligonucleotides and conjugation inhibitors because they represent good examples of alternative strategies against AMR.

### 8.1. Oligonucleotides

The application of oligonucleotides to modulate the expression of proteins related to virulence and drug resistance mechanisms on microbes has attracted increasing attention in the last decades. Unfortunately, oligonucleotide administration has some drawbacks related to their low stability and difficulties in delivering into bacteria. Oligonucleotides are easily degraded by exo- and endonucleases present in many biological tissues, fluids, or biofilms. Therefore, their protection and stabilization are essential to achieve their therapeutic effect. Moreover, bacterial cell wall architecture, biofilms or access to intracellular pathogens hinder the efficient delivery of oligonucleotides to target sites where the oligonucleotides exert their regulatory function [130].

In this regard, developing proper DDSs for oligonucleotides administration is an innovative research field to improve the stability, selective delivery of antisense oligonucleotides [130], or combined delivery for oligonucleotides and ribonucleoproteins, as in the case of CRISPR/Cas technology [131].

Different groups have demonstrated the ability of SLNs to efficiently deliver oligonucleotides for several applications. For instance, cationic SLNs have been applied for the co-delivery of an antitumoral drug and mi-RNA to glioblastoma cells obtaining a significant increase in cell uptake [132]. In another interesting approach, cationic SLNs were loaded with decoy oligodeoxynucleotides to block STAT3 in ovarian cancer cells obtaining a potent induction of cell death and cell invasion inhibition [133]. Antisense oligonucleotides have also been loaded into SLNs to reduce the intracellular activity of SMAD3, obtaining a significant anti-inflammatory effect [134].

Unfortunately, to our knowledge, only one work has been published about the delivery of oligonucleotides for antibiotic purposes. In a previously mentioned interesting work, SLNs were formulated to deliver oligonucleotides to modulate transcription factor decoy (TFD) expression in two different bacteria: SigH, which specifically blocks the key sigma factor of the transition phase in *Clostridium difficile*, and the ferric uptake regulator (Fur), which controls the import of the essential micronutrient iron into *Escherichia coli* under limiting conditions [50]. Three different cationic SLNs formulations were developed and characterized to achieve optimal oligonucleotide delivery. They demonstrated that oligonucleotide-loaded SLNs could deliver TFD to cells with high efficiency and to obtain a specific antibacterial activity, showing a good safety profile in eukaryotic cells at the concentrations needed for antibacterial activity.

Recently, CRISPR/Cas technology has attracted the attention of many scientists for the possible application of this technology in the treatment of many complex diseases. This gene-editing technology has the potential to very selectively insert, delete, or mutate genes in almost any species, including bacteria. Several researchers have applied this technology as a selective antibacterial weapon obtaining promising results against resistant bacterial strains [135,136,137]. Nonetheless, applying this technology to reduce antibiotic resistance mechanisms is still in progress, as many details must be considered. For instance, it requires advances in delivery and targeting specific pathogenic bacteria within complex bacterial populations, such as our gut microbiome, delivering antibacterials to pathogenic bacteria, or bacteria-infected host cells [138]. Therefore, selective delivery of the CRISPR/Cas system can be as important as its own editing activity. Delivery of the CRISPR/Cas system can be performed in viral or non-viral vectors. Since viral vectors, although effective, must be subject to many biosafety considerations, non-viral vectors may represent a very promising alternative in this field. In this regard, lipid nanoparticles have the potential to efficiently deliver CRISPR/Cas system into the target cells [131,139]. Thus, applying CRISPR/Cas technology is a new promising strategy to fight against AMR, provided proper DDSs are developed, and in this regard, SLNs could be an interesting alternative to viral vectors. Nonetheless, this possibility is undeveloped and requires further research.

### 8.2. Conjugation Inhibitors

**Figure 6:**
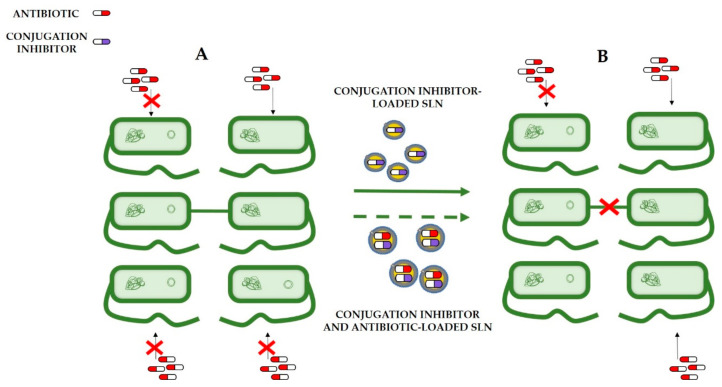
Schematic representation of (**A**) antibiotic resistance spreading by bacterial conjugation. At the beginning of the process, the bacterium at the left is resistant to antibiotics, but the right is not. After conjugation, both bacteria are resistant because they have shared the resistance genes coded in the plasmid (circular DNA). (**B**) Antibiotic resistance spreading inhibition (continuous line) and antibiotic resistance spreading inhibition in combination with antibiotic treatment (dashed line). Administration of conjugation inhibitors loaded into SLNs can prevent the conjugation process, avoiding resistant gene spreading. Additionally, the administration of conjugation inhibitors and antibiotics in the same SLNs treatment of the infection and inhibition of antibiotic resistance spread could be achieved.

Bacteria can share genetic material via HGT, disseminating resistance genes and facilitating the appearance of MDR bacterial strains. Thus, inhibition of this process could help to decelerate developing the global health crisis, reducing the spreading of antibiotic resistance genes among bacteria. As mentioned before, conjugation is one of the main mechanisms by which bacteria can acquire genetic material, and that is why inhibiting conjugation can be an interesting weapon against drug resistance. Nevertheless, these inhibitors should not target essential bacterial functions to avoid developing new resistance mechanisms. Different conjugation inhibitors have been developed to attack specific components of the conjugation machinery, such as VirB8-like proteins and VirB11-like proteins (one of the ATPases of the T4SS) [12], relaxase inhibitors or pilus blockers [140]. A combination of these inhibitors with antibiotics can be a potent weapon against antimicrobial-resistant bacteria [140]. In this context, applying SLNs for combined drug delivery seems an interesting strategy (Figure 6).

Of particular interest would be applying SLNs to incorporate unsaturated fatty acids that have been reported as interesting conjugation-inhibitors of VirB11-like protein of R388 plasmid, TrwD_R388_ [141,142,143]. Oleic acid, linoleic acid, dehydrocrepenynic acid, tanzawaic acid or some synthetic 2-alkynoic fatty acid derivatives present potential conjugation inhibition activities, but their structure and reduced solubility impair their administration as drugs. Considering the physicochemical characteristics of these bioactive lipids, incorporation of these unsaturated fatty acids into SLNs would solve the solubility and administration problems. Furthermore, SLNs composed of these bioactive lipids can be combined with other antibiotic drugs to attain potent treatment against antimicrobial infections.

## 9. Conclusions and Future Perspectives

Antimicrobial resistance is currently one of the biggest threats to global health, and we urgently need new strategies to tackle it. Considering the drawbacks that developing new antibacterial drugs represents, the improvement of the efficiency of well-known drugs by nanocarriers seems the most promising approach.

In this context, solid lipid nanoparticles offer highly interesting opportunities to increase antibiotic efficiency and reverse or reduce antibiotic resistance. SLNs can improve drug stability, solubility and permeability, enhancing its bioavailability and, most importantly, drug concentration in the target site. Moreover, they have demonstrated their ability to overcome some of the basic resistance mechanisms developed by resistant bacterial strains, such as reduced drug permeation, intracellular infections or biofilm formation. Additionally, some other mechanistic advantages, such as protection against enzymatic drug degradation or inhibition of efflux pumps, should be further studied.

Apart from the above-mentioned advantages, SLNs provide an encouraging approach for a drug combination strategy, facilitating the spatial-temporal distribution of all administrated molecules. The combination strategy limits bacterial resistance as the additive or synergistic effect of more than one active molecule reverses the resilience of the resistant strains.

Finally, applying SLNs can open the spectrum of possible bioactive molecules that do not present good stability, solubility or permeability and that, without their incorporation into a DDSs, could not be used as antibacterial molecules. Thus, SLNs application broadens the spectrum of new possible antibiotic agents.

Despite being described for the first time more than twenty years ago and having all their advantages demonstrated, there are still many questions to answer about applying SLNs as DDSs. The relation between SLNs characteristics and their biological behavior regarding bacterial infections is still unclear, possibly because few works have been performed to study the specific interactions between SLNs and bacteria. One reason to explain this lack of information could be that sometimes it is difficult to observe intracellular drug fate or interactions between SLNs and bacterial cell walls. Moreover, many studies are centered on the final efficiency of the therapy, but very few works look beneath it to elucidate cellular mechanisms implicated in those processes. These unanswered questions difficult the translation of basic research into clinical trials, which could be why there are still few trials with SLNs applications. Nonetheless, the urgency of new antimicrobial treatments deserves a global effort to solve these questions and to improve SLNs’ characteristics against antibiotic resistance dissemination.

## Figures and Tables

**Table 1:** Characteristics of SLNs formulations applied to reduce resistance mechanisms.

	Drug	Size	pdi	Zeta-Potential (mV)	EE (%)	Efficiency Enhancement	Organism	Ref.
Efflux pumps	Fluconazole	84.8 ± 4.2	0.291 ± 0.012	−25 ± 4.1	89.6 ± 3.97	Avoidance of drug recognition by efflux pump proteins	*Candida glabrata*	[91]
	Rifampin	108.7 ± 5.5	0.18	−10.7 ± 0.5	82	Reduction of drug expulsion	*Mycobacterium fortuitum* (ATCC 2701P)	[92]
Infections by intracellular pathogens	Rifampicin	440 ± 40	0.37 ± 0.01	− 49.73 ± 0.50	52.45	Relevant and significant increase in drug content within the macrophage; Uptake by macrophages involving mannose receptors	J774A.1	[55]
	Enrofloxacin; docosanoic acid; 2% PVA; dimethyldioctadecyl ammonium chloride (0.5–4%)	414.5 ± 3.8; 617.5 ± 7.1; 532.1 ± 10.0; 501.3 ± 16.6; 345.2 ± 9.6	0.265 ± 0.019; 0.458 ± 0.010; 0.461 ± 0.058; 0.417 ± 0.016; 0.393 ± 0.011	−22.1 ± 0.1; −17.5 ± 0.6; −8.1 ± 0.4; 7.1 ± 0.5; 18.8 ± 0.2	86.6 ± 1.7; 42.8 ± 2.3; 41.2 ± 0.8; 46.7 ± 2.4; 45.6 ± 1.8	Enhanced cellular uptake; Slower elimination of enrofloxacin after removing extracellular drug; Stronger inhibition effect against intracellular *Salmonella* CVCC541	Intracellular *Salmonella* CVCC541	[56]
	Doxycycline	299 ± 34	0.29 ± 0.027	−28.7 ± 3.2	94.9 ± 3.2	Reduced number of bacteria inside J444A.1 macrophages	Intracellular *Brucella melitensis*	[99]
	isoniazid	236 ± 9	0.240 ± 0.012	− 19 ± 2	75.13 ± 0.97	Increased intracellular antibiotic efficiency for the in vitro latent tuberculosis infection model; Superior antibiotic efficacy in the in vivo antibiotic tests compared to the INH solution	*Mycobacterium tuberculosis*; Wistar rats	[100]
	Enrofloxacin	341.4 ± 4.9; 348.8 ± 3.5; 408.5 ± 6.3; 414.5±3.8	0.241 ± 0.014; 0.264 ± 0.013; 0.352 ± 0.015; 0.265±0.019	−19.9 ± 0.4; −20.6 ± 0.9; −21.3 ± 0.6; −22.1 ± 0.1	65.2 ± 1.76; 67.53 ± 2.25; 72.57 ± 2.90; 86.56±1.60	Enhanced cellular uptake; Slower elimination of enrofloxacin after removing extracellular drug; Stronger inhibition effect against intracellular *Salmonella* CVCC541	Intracellular *Salmonella* CVCC541	[90]
	Rifabutin-uncoatedrifabutin–mannose	389 ± 2.3; 251 ± 5.1	0.357; 0.439	3.38 ± 0.3; −11.7 ± 0.8	87.8 ± 1.2; 82.6 ± 1.2	Mannosylation enhances macrophage uptake Mannosylation promotes; selective uptake by lung tissues	J774 macrophages; Healthy albino rats	[103]
Biofilm formation and quorum sensing	Cefuroxime axetil	279.2 ± 28.5	0.107 ± 0.07	−23.58	70.62 ± 0.82	Drug minimum biofilm inhibitory concentration is 50% lower in SLNs	*Staphylococcus aureus* (ATCC-25923	[105]
	Rifampin	101.7 ± 4.7	0.284 ± 0.024	+17.1 ± 0.7	69% ± 2.1	Significant reduction of the viability of bacteria embedded in biofilms	Biofilm-producing *Staphylococcus epidermidis*	[106]
	Clarithromycin	307 ± 23	0.21 ± 0.04	−29.0	84 ± 9	Enhanced in vitro antibacterial activity; Higher potential in biofilm eradication compared to free drugs; Almost 5-fold improvement in relative oral bioavailability	*Staphylococcus aureus*; (MTCC86)Wistar rats	[107]
	Curcumin	423.7 ± 23.2	0.310 ± 0.076	−25.9 ± 6.7	85	Satisfactory inhibition of biofilms	*Staphylococcus aureus*; (ATCC-12600)	[108]
	Colistin sulfate	300–427	0.3–0.4	n.d.	80–95	Efficient eradication of biofilms	*Pseudomonas aeruginosa*	[109]
	Tobramycin	302 ± 20.5	0.361 ± 0.02	−20.5 ± 6.09	n.d.	Increased biofilms eradication	*Pseudomonas aeruginosa*	[110]
	Quorum sensing inhibitor (2-heptyl-6-nitro-4-oxo-1,4-dihydroquinoline-3-carboxamide)	<100 nm	<0.2	−(15–35)	Reduction in pyocyanin 73.4	(virulence factor) formation; High deposition in the bronchial area, the target site	*Pseudomonas aeruginosa*; Calu-3 cells	[112]

n.d.: not defined in the paper.

**Table 2:** Characteristics of SLNs formulations for combined therapy.

Drug	Size	pdi	Zeta-Potential (mV)	EE (%)	Efficiency Enhancement	Organism	Ref.
Ampicillin and; curcumin	163 nm	<0.5	n.d.	n.d.	Overcome resistance to free antibiotic; Overcome resistance to free antibiotic; Minimum bactericidal concentration decreased 4 times comparing to free drugs; Resistance of bacteria to free drugs is broken	*Bacillus subtilis*; *Pseudomonas aeruginosa*; *Corynebacterium diphtheriae*; Methicillin-resistant *Staphylococcus aureus*	[121]
Chitosan + ofloxacin/eugenol	210.1 ± 5.9	0.418 ± 0.033	15.47 ± 0.21	Ofloxacin 33.5 ± 1.9	Minimum inhibitory concentration six-fold lower concerning the free antibiotic; MIC 16-times lower than that of free drug	*Pseudomonas aeruginosa*; *Staphylococcus aureus*	[122]
Rifampin + cis-2-decenoic acid	127.2 ± 2.8	0.263 ± 0.017	19.0 ± 7.64	Rifampin 69 ± 5.10C2DA 46 ± 4.23	In vitro anti-biofilm activities at both formation and eradication stages	*Staphylococcus aureus*; *Staphylococcus epidermidis*	[123]
Ampicillin + nisin Z	175.457± 17.885	0.279 ± 0.057	−42.078 ± 0.903	Ampicillin 43.826 ± 4.596	Selective toxicity toward bacterial cells; Enhanced antibacterial activity of nisin ZNo improvement (electrostatic problems)	*Staphylococcus aureus*; *Staphylococcus epidermidis* *Escherichia coli*	[124]
Clotrimazole–Ag	124.1± 2.5	0.235 ± 0.02	−30.3 ± 5.9	CTM 96.94 ± 0.42	Enhanced and sustained antibacterial activity	Methicillin-resistant *Staphylococcus aureus* Methicillin-susceptible *Staphylococcus aureus*	[125]
Ciprofloxacin–selenium	153.6 ±1.8	0.134 ± 0.03	−1.74 ± 0.27	CPF 40.4±4.4	Greater antibacterial activity; Prevented the liver tissue damage	*Pseudomonas aeruginosa*; Mice	[126]
LL37 + serpin A1	214.9 ± 2.2; 261.7 ± 4.4	n.d.; n.d.	−20 ± 1.8; −21 ± 2.1	LL37:84.8 ± 2.7 A1:87 ± 3.5; LL37:81.6 ± 3.2A1:83.3 ± 4.1	Synergistically enhance the antibacterial activity; In vitro accelerated wound healing	*Staphylococcus aureus, Escherichia coli*; Fibroblast and keratinocytes	[127]
Levofloxacin + DNase	162.9 ± 5.3	0.340 ± 0.014	−10.3 ± 0.3	Levo 55.9 ± 1.6%	A strong antibacterial activity (less than free drug); Destroy biofilms after 24 h (*Pseudomonas aeruginosa*)	* Pseudomonas aeruginosa * *Staphylococcus aureus*	[128]
Anacardic acid + chitosan + DNAse	212.8 ± 4.21	0.285 ± 0.04	+13.5 ± 1.92	Ana 73.8 ± 1.23%	Higher biofilm eradication activity	*Staphylococcus aureus*	[129]

n.d.: not defined in the paper.
